# SIRT5: a potential target for discovering bioactive natural products

**DOI:** 10.1007/s11418-024-01871-6

**Published:** 2025-02-20

**Authors:** Yuwei Xie, Nali Cai, Xiaohua Liu, Liangliang He, Yiming Ma, Changyu Yan, Juan Liang, Shu-Hua Ouyang, Ao Luo, Yingzhi He, Jun Lu, Dang Ao, Jia Liu, Zhonglv Ye, Bin Liu, Rong-Rong He, Wen Li

**Affiliations:** 1 Department of Pediatrics, Affiliated Hospital of Guangdong Medical University, Zhanjiang, 524001 China; 2 Guangdong Engineering Research Center of Traditional Chinese Medicine & Disease Susceptibility, Jinan University, Guangzhou, 510632 China; 3 Laboratory of Hepatobiliary Surgery, Affiliated Hospital of Guangdong Medical University, Zhanjiang, 524001 China

**Keywords:** SIRT5, Substrate, Deacetylation, Desuccinylation, Demalonylation, Deglutarylation

## Abstract

Silent information regulator 5 (SIRT5) is the fifth member of the sirtuin family, which is mainly expressed in mitochondrial matrix. SIRT5 plays a key role in metabolism and antioxidant responses, and is an important regulator for maintaining intracellular homeostasis. Given its involvement in multiple cellular processes, dysregulation of SIRT5 activity is associated with a variety of diseases. This review explores the structural characteristics of SIRT5 that influence its substrate specificity, highlights recent research advances, and summarizes its four key enzymatic activities along with their corresponding substrates in disease contexts. We also discuss the natural products that modulate SIRT5 activity and identify potential targets of SIRT5 through virtual docking, which may provide new therapeutic avenues. Although the mechanism of SIRT5 in diseases needs to be further elucidated and deglutathionylation activities are still at an early stage, targeting SIRT5 and its substrates holds significant promise for the development of novel therapeutics.

**Graphical abstract:**

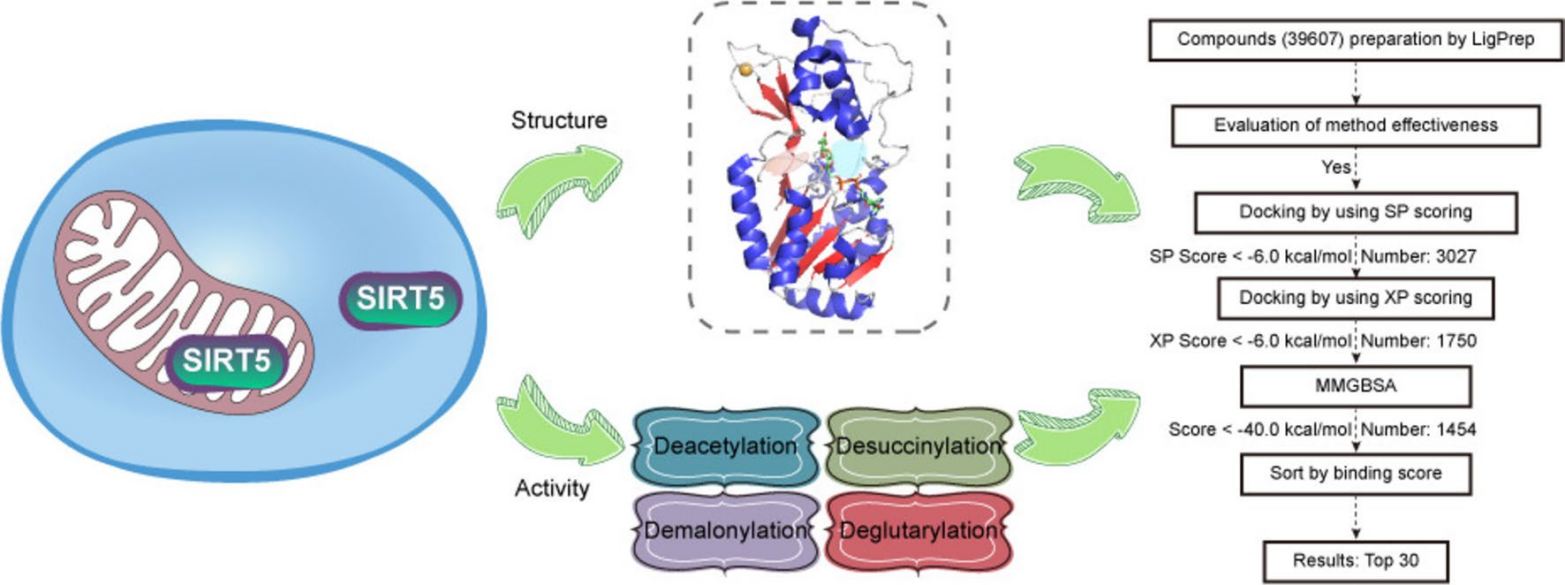

**Supplementary Information:**

The online version contains supplementary material available at 10.1007/s11418-024-01871-6.

## Introduction

**Fig. 1:**
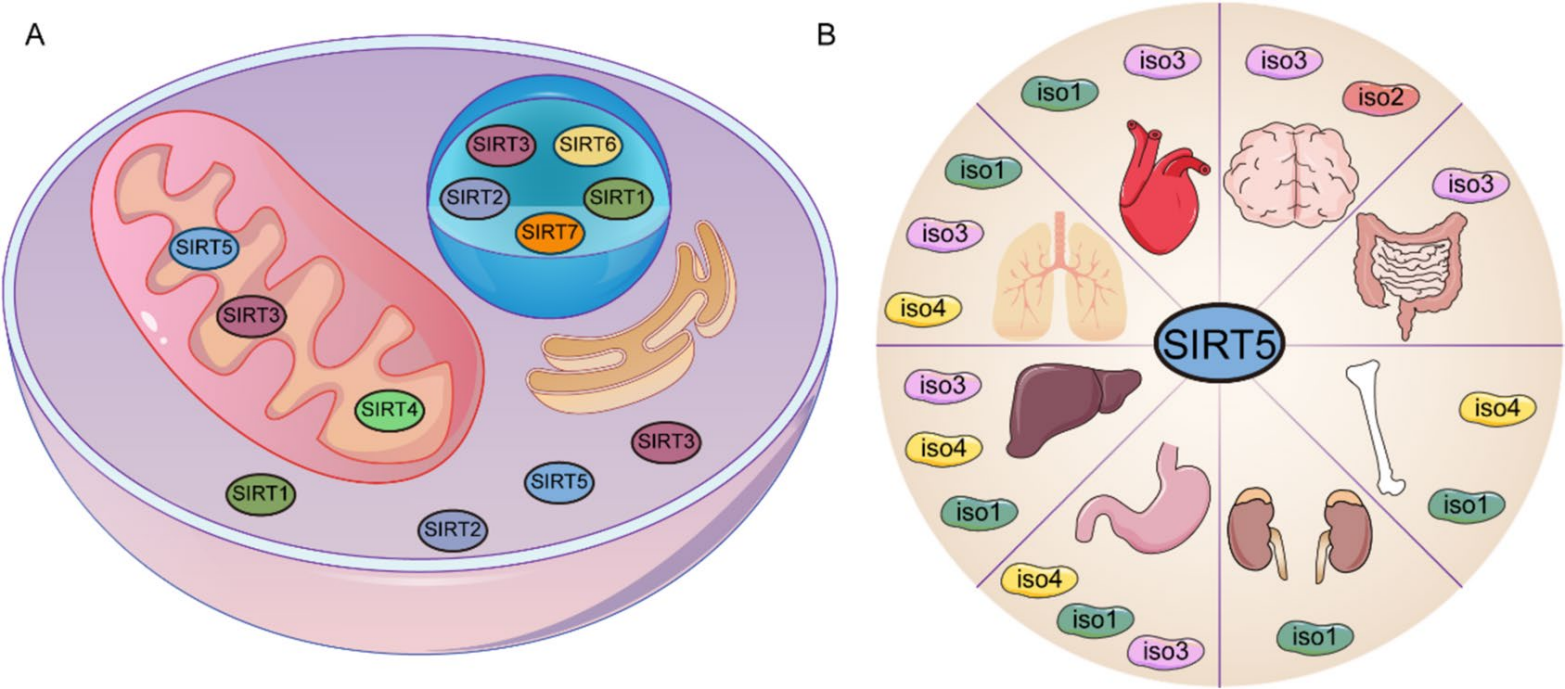
Distribution of sirtuins and SIRT5 isoforms. **A** Subcellular localization of sirtuins. SIRT1 is primarily expressed in the nucleus and to a lesser extent in the cytoplasm. SIRT2 is found both in the cytoplasm and the nucleus. SIRT3-5 are mainly located in the mitochondria but also expressed in the cytoplasm and the nucleus. SIRT6-7 are distributed only in the nucleus. **B** Tissue expression of SIRT5 isoforms. SIRT5^iso1^ is mainly enriched in kidney, lung, stomach, liver, and heart. SIRT5^iso2^ is mainly expressed in brain. SIRT5^iso3^ is highly exhibited in brain, intestine, lung, stomach, liver, and heart. SIIRT5^iso4^ is mainly distributed in lung, stomach, and liver

Silent information regulator 2 (SIR2) was discovered when researchers studied the lifespan of *Saccharomyces cerevisiae* [1], which opened the research of sirtuins protein family and its biological properties [2]. Unlike the zinc-dependent histone deacetylases (HDACs) of Class I, II and IV, the class III HDACs are nicotinamide adenine dinucleotide (NAD^+^)-dependent, also known as sirtuins. Sirtuins participate in the pathophysiological process of diseases through protein post-translational modification (PTMs) [3]. Seven mammalian homologues of sirtuins (Sirtuin1-7) have been identified, with different subcellular localization and function. SIRT1 is mainly located in the nucleus and less expressed in the cytoplasm. SIRT2 can be found both in the cytoplasm and nucleus. SIRT3-5 are mainly located in the mitochondria but also expressed in the cytoplasm and nucleus. SIRT6-7 only distribute in nucleus [4] (Fig. 1A).

Recently, SIRT5 has received extensive attention due to its specific catalytic activity for weak deacetylation activity but strong desuccinylation, demalonylation, and deglutarylation activities [5]. SIRT5 is a key regulator of various physiological processes such as cellular differentiation, apoptosis, metabolism, aging, immune response, oxidative stress, and mitochondrial function. Therefore, SIRT5 is also considered as a promising drug target for treatment of related diseases. Indeed, due to the abundance of hydrogen bond donors and acceptors in the structure of SIRT5, the compounds described in the paper are likely to be pleiotropic and target multiple pathways, not just those mentioned in the paper. In particular, SIRT5 plays a dual role in tumor. Studies have shown that SIRT5 is highly expressed in non-small cell lung cancer (NSCLC), and inhibition of SUN2 promotes cancer cell proliferation [6]. However, it has also been reported that SIRT5 is downregulated in NSCLC, allowing STAT3 to be acetylated, thereby promoting ATP synthesis and supporting cell growth [7]. Therefore, more studies on SIRT5 are needed to facilitate the discovery of a selective regulatory compound. Investigating the selective regulation of SIRT5 will not only contribute to further understanding of its role in physiological and pathological settings, but also has the potential to be applied to the clinical treatment of specific pathologies.

In this paper, we highlight the unique structure and catalytic mechanism of SIRT5, summarize the four enzymatic activities and their corresponding targets in association with diseases. Finally, we summarize natural compounds that regulate SIRT5 activity either as activators or inhibitors and discuss the mining of SIRT5 regulatory compounds.

## Localization and expression of SIRT5

The human *Sirt5* gene is located on band 3 of short arm 2 of chromosome 6, spanning a region of 28,182 bp, including 8 exons ranging in size from 54 to 226 bp. The short interspersed nuclear elements (SINEs) and the long interspersed nuclear elements (LINEs) are clustered in introns 1, 2, 5 and 7, whereas the catalytic domain of sirtuin is located in exons 2 and 7, between amino acid residues 54 and 256 [8]. The *Sirt5* gene encodes four protein isoforms, namely SIRT5^iso1^, SIRT5^iso2^, SIRT5^iso3^ and SIRT5^iso4^, with a molecular weight of about 34 kDa. SIRT5^iso1−3^ were mitochondria-localized, while SIRT5^iso4^ were mainly localized in the cytoplasm [9]. The gene encoding SIRT5 is widely expressed in tissues, but different isoforms have different expression patterns. SIRT5^iso1^ is expressed in almost all human tissues, except adipose, bladder, and trachea, with brain and kidney being the most abundant. SIRT5^iso2^ is most frequently discovered in the brain, but less in other tissues. SIRT5^iso3−4^ are mainly expressed in brain and kidney [9] (Fig. 1B).

## Crystal structure of SIRT5

**Fig. 2:**
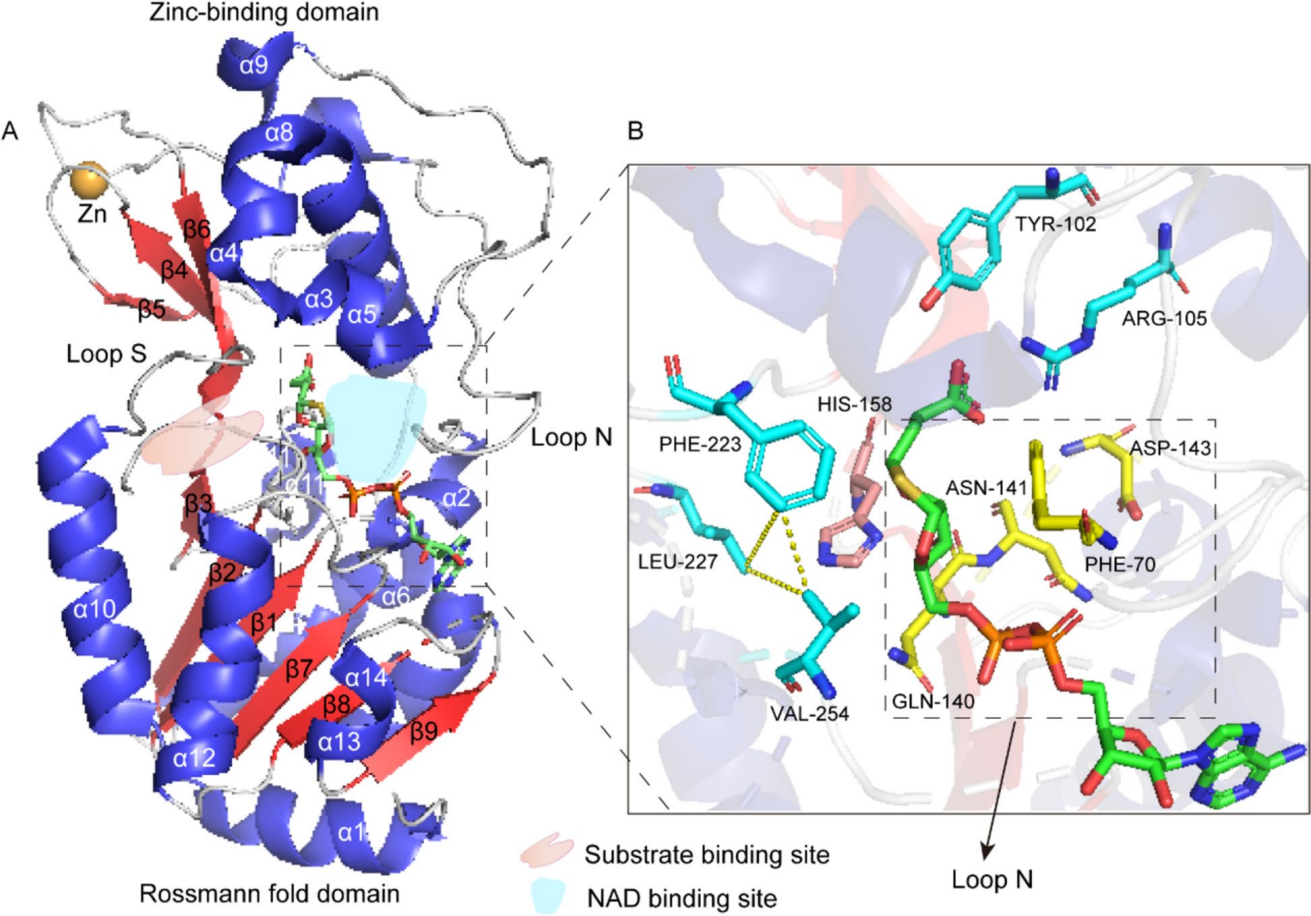
Crystal structure of SIRT5. **A** SIRT5 includes a zinc finger binding domain and a Rossmann fold domain. Substrate-binding site and NAD^+^ binding site are formed between these two domains, where Loop S binds to substrate and Loop N binds to NAD^+^. **B** Amplify the binding site of SIRT5 to focus on the interaction of substrate peptides and NAD^+^ with SIRT5 residues. Phe70, Gln140, Asn141, and Asp143 residues play an important role in binding to NAD^+^, while Phe223, Leu227, and Val254 residues form a small triangle, which is the recognition entry for acyl-lysine. Besides, Tyr102, Arg105, and His158 residues directly interact with the acyl-lysine substrate

The crystal structure of SIRT5 is composed of a zinc finger binding domain and a Rossmann fold domain, with a total of 14 α-helices and 9 β-strands. Among them, 3 antiparallel β-strands (β4, β5 and β6) and 5α-helices (α3, α4, α5, α8 and α9) constitute the zinc finger binding domain, while the Rossmann fold domain consists of 6 parallel β-strands (β1, β2, β3, β7, β8 and β9) and 9α-helices (α1, α2, α6, α7, α10, α11, α12, α13 and α14) [10]. A substrate-binding site and a NAD^+^ binding site are formed between the two domains. One is called loop S, a large flexible loop formed by the β6 of the zinc finger domain and α10 of the Rossmann fold domain, which binds to the substrate and achieves a structural conformational change [11]. In loop S, the hydrophobic residues Phe223, Leu227, and Val254 form a small triangle, which is the recognition entrance of acyl-lysine [12]. The other is loop N, a loop formed by β3 of the zinc finger domain and α2 of the Rossmann fold domain that binds to NAD^+^ [13]. In SIRT5, Phe70, Gln140, Asn141, and Asp143 form the loop N. Gln140 and Asn141 bind to NAD^+^ ribose. Asp143 binds to nicotinamide. Phe70 has a role in binding NAD^+^ and releasing nicotinamide [13] (Fig. 2A).

SIRT5 has some of the same structure as other sirtuins. For instance, SIRT1-3 also have a zinc finger domain consisting of 5α-helices and 3 antiparallel β-strands. Moreover, the recognition entrance of acyl-lysine is also composed of Phe223, Leu227, and Val 254 and positioned in the same locations [14–16]. However, the structure of SIRT5 is unique: the deep end of the substrate-binding pocket consists of two non-hydrophobic residues, Tyr102 and Arg105, which recognize the negatively charged acyl-lysine groups through hydrogen bonding and electrostatic interactions [17]. Changes in Arg105 to Met or Tyr102 to Phe significantly increased the *K*_m_ for desuccinylation [13]. Consequently, these two amino acids are essential for the enzymatic activity of SIRT5. In addition, other sirtuins contain Phe223 residues in their substrate-binding pockets, which prevent the entry of large groups to ensure substrate selectivity. In contrast, the residue Ala86 in SIRT5, rather than Phe223, makes the pocket larger and thus allows entry of larger acylated lysine substrates. These amino acids contribute to the structural specificity of SIRT5 and determine its substrate selectivity and enzymatic activity (Fig. 2B).

As we all know that sirtuins have thousands of target proteins, here we only cite some examples of them that have been well-studied and elucidated their mechanism, in the hope that it will inspire the readers. The localization and expression, enzymatic activities, well-studied targets and biological functions of sirtuins family proteins are summarized in Table 1.

**Table 1:** Summary of localization and expression, enzymatic activities, well-studied targets and functions of mammalian sirtuins family proteins

Protein	Location	Expression	Activity	Target	Function	References
SIRT1	Nucleus /cytoplasm	Brain, adipose tissue, heart, kidney, liver, retina, skeletal muscle, blood vessels, uterus	Deacetylase	p53, HIC1	Regulate cellular senescence	[18, 19]
				FOXO3, FOXO1, NF-κB, E2F1, Ku70	Control apoptosis, cell cycle progression, DNA repair	[20–24]
				PCK1, HIF1α	Glucose metabolism	[25, 26]
SIRT2	Cytoplasm /nucleus	Adipose tissue, brain, heart, kidney, liver, skeletal muscle, blood vessels	Deacetylase	Histone H4, CDC20	Cell cycle control	[27, 28]
				Histone H3	Anti-infection	[29]
				PAR-3	Promote nerve cell development	[30]
				G6PD, FOXO1	Regulates redox reactions	[31, 32]
				HIF1α	Regulate metabolism	[33]
SIRT3	Mitochondria/nucleus /cytoplasm	Adipose tissue, brain, heart, kidney, liver, oocyte, skeletal muscle, blood vessels	Deacetylase	AceCS2, OSCP, FOXO3	Regulate of metabolism	[34–36]
				LCAD	Regulate fatty acid oxidation	[37]
SIRT4	Mitochondria	Brain, heart, kidney, liver, blood vessels, pancreatic beta cells	ADP-ribosyltransferase	GDH	Insulin secretion	[38]
			Delipoamidase	PDH	Regulation of mitochondrial metabolism	[39]
			Deacetylase	MCD	Fat oxidation	[40]
			HMG-deacylase	HMG and related acyl	Insulin secretion	[41, 42]
SIRT5	Mitochondria/cytoplasm	Brain, heart, kidney, liver, blood vessels, thymus, testis, skeletal muscle	Deacetylase	CPS1	Regulate urea cycle	[43]
			Desuccinylase	ECHA	Regulate heart metabolism and function	[44]
				PDHA1	Suppress ccRCC	[45]
			Demalonylase	GAPDH	Regulate glycolysis	[46]
			Deglutarylase	G6PD	Regulate NADPH homeostasis and redox potential	[47]
SIRT6	Nucleus	Brain, heart, kidney, liver, blood vessels, retina, skeletal muscle, thymus, testis, ovary	deacetylase	FOXO1	Glucose reabsorption and gluconeogenesis	[48]
				Histone H3	Telomere stability, transcriptional regulation, DNA damage response	[49]
			ADP-ribosyltransferase	PARP1	Promote DSBs repair under stress	[50]
			Defatty-acylation	TNF-α	Regulation of TNFα secretion	[51]
SIRT7	Nucleus	Heart, blood vessels, liver, brain, skeletal muscle, peripheral blood cells, spleen, testes	Deacetylase	Histone H3	Transcriptional repression	[52]
				FBL	Cell cycle regulation	[53]
			Desuccinylation	Histone H3	Promoting chromatin condensation and DSB repair	[54]
			Decrotonylation	PHF5A	Regulate aging	[55]
			ADP-ribosyltransferase	ADP	Regulate glucose homeostasis and aging	[56]

## Binding characteristics of SIRT5-targeted substrates

As mentioned above, the unique biological structure of SIRT5 is the basis for its targeting of specific substrates. Acetylated groups, succinyl groups, malonylated groups, and glutarylated groups in the peptide chain are targets of SIRT5 deacylation [57–59].

## Deacetylation by SIRT5

Sirtuins contain a conserved enzymatic core consisting of a Rossmann fold domain and a small domain formed by two insertions within the Rossmann fold [60]. The Rossmann fold domain and the small domain are separated by gaps that bind by NAD^+^ and acetylated peptide substrates [10]. Hydrolysis of glycosidic bonds between nicotinamide and ribose forms *O*-alkylamide acid intermediates [61]. Protons are then extracted and donated to the intermediate to complete the reaction, resulting in the release of deacetyllysine, nicotinamide, and 2’-*O*-acetyl-ADP-ribose (which can be esterified into 3’-*O*-acetyl-ADP-ribose) [62].

**Fig. 3:**
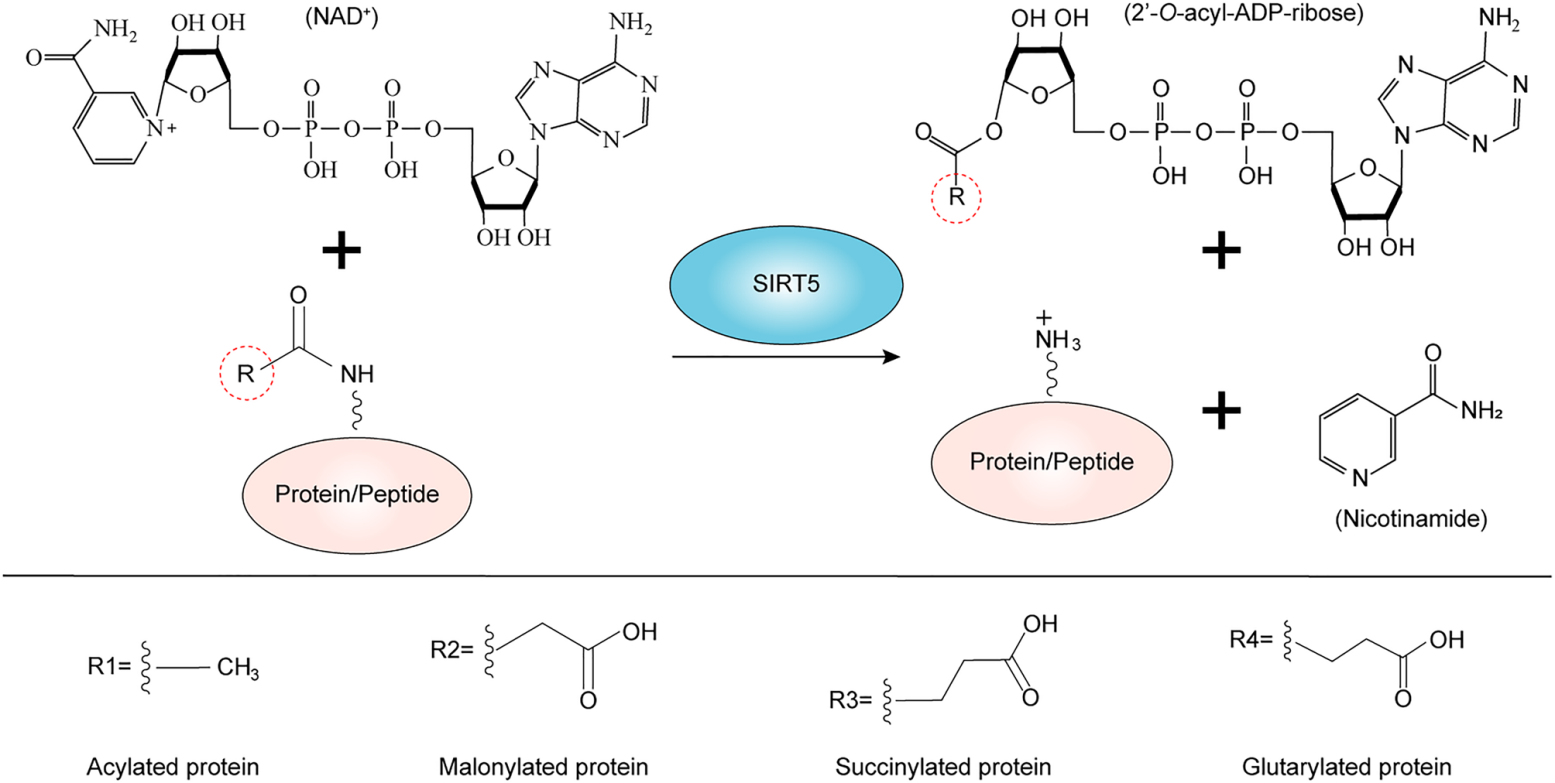
SIRT5-catalyzed deacylation reaction. Acylated protein (including acetylated, succinylated, malonylated and glutarylated proteins) and NAD^+^ are catalyzed by SIRT5 to produce corresponding 2’-*O*-acyl-ADP-ribose, nicotinamide, and deacylated protein. The R group can be replaced by an acetylated group, a succinylated group, a malonylated group, or a glutarylated group

Specifically, when nicotinamide begins to dissociate, on the one hand, carbonyl oxygen is attracted to the C10 position due to electrophilic reaction. On the other hand, the conformation of the ribose ring changes, bringing C10 closer to the carbonyl group. These effects jointly promote the formation of *O*-alkyl ester intermediates. Once an intermediate is formed, the invariant active site histidine (His158) extracts a proton from the 2’ or 3’ hydroxyl group to activate the 2’-OH of *N*-ribose, leading to the formation of the final reaction product. This is a base exchange reaction that alters NAD^+^ at the expense of deacetylation [62] (Fig. 3).

## Desuccinylation by SIRT5

The structure of SIRT5-bicyclic intermediate has been confirmed in the deacetylation mechanism of sirtuins, and thioacetyl-lysine peptides have been identified as sirtuins inhibitors with deacetylase activity and used in Sir2Tm deacetylation studies [13]. Biochemical studies showed that the mechanism of SIRT5-catalyzed desuccinylation and demalonylation was similar to that of deacetylation catalyzed by sirtuins [12]. Based on this background, a thiosuccinyl-lysine peptide was developed as a SIRT5-specific inhibitor to understand SIRT5-catalyzed desuccinylation [63].

Succinyl peptide is specifically recognized by Tyr102 and Arg105, catalyzing the formation of hydrogen bonds between His-158 and the 3’-OH of *N*-ribose. The nicotinamide group of NAD forms hydrogen bonds with the side chain of Asp143 and the main chain nitrogen of Ile-142, while the conformation of NAD changes to make room for the release of nicotinamide. In addition, Gln140 and Asn141 form hydrogen bonds with 3’-OH and 2’-OH of *N*-ribose, respectively. Together, these interactions promote cleavage of nicotinamide. The carboxyl oxygen of the succinyl group then forms with the 3’-OH of ribose to form a bicyclic intermediate, which is further hydrolyzed to free lysine and succinyl-*O*-ADPR [12].

## Demalonylation by SIRT5

PTMs are an important way to regulate protein function and participate in many physiological and pathological processes. Since the malonylation of lysine was discovered, researchers have been found that SIRT5 catalyzes the demalonylation of lysine [57]. However, the exact mechanism is unclear. Based on the structure of SIRT5, it was hypothesized that the mechanism of demalonylation is similar to deacetylation, and this hypothesis was confirmed by ^32^P-NAD mass spectrometry detection and the formation of malonyl-*O*-ADPR [13].

## Deglutarylation by SIRT5

Glutaryl-CoA is an important metabolite of amino acid metabolism, which is structurally similar to succinyl-CoA and malonyl-CoA. At physiological pH, glutaryl-modified lysine residues are negatively charged while there are two positively charged amino acids (Tyr102 and Arg105) at the active center of SIRT5. When the substrate enters the deep end through entry recognition, the acyl group in the substrate peptide chain must be negatively charged to be specifically recognized and bound by Tyr102 and Arg105. The two substrates, NAD^+^ and lysine, enter the active site from opposite sides, and the lysine-carrying peptides occupy the cracks between these two domains [64]. The substrate lysine interacts with NAD^+^, leading to the conformation of NAD^+^ changes to release nicotinamide. The rotation of *N*-ribose promotes the nucleophilic attack on the carboxyl oxygen of the acyl group, thereby forming the ADPR-peptide amide intermediates, which are further hydrolyzed to generate free lysine and peptidyl-*O*-ADPR [65] (Fig. 3).

## The biological functions of SIRT5 enzyme activities

**Fig. 4:**
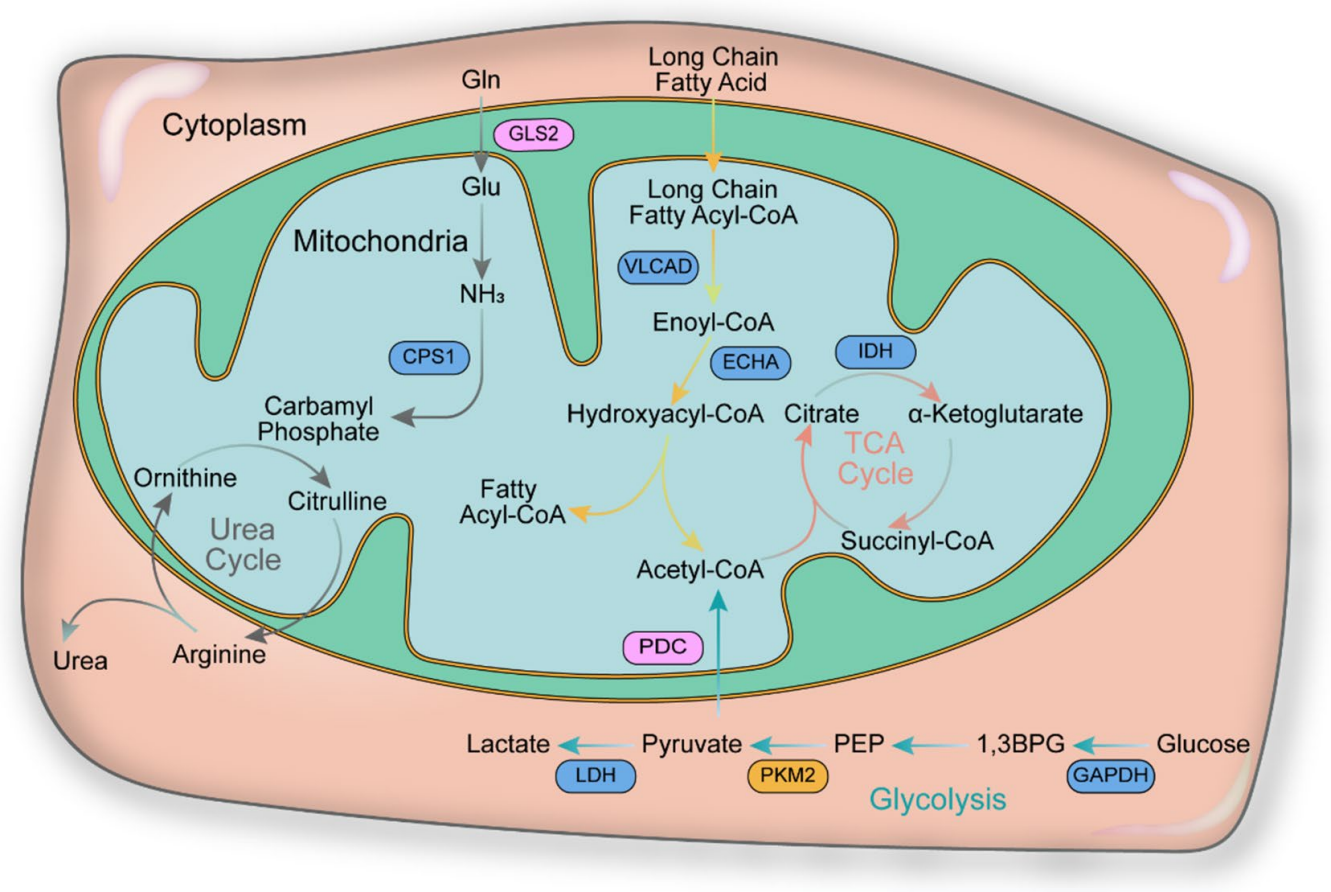
SIRT5 regulates four major metabolic pathways: urea cycle, fatty acid β-oxidation, TCA cycle and glycolysis. Targets in the blue frames are activated by SIRT5, and targets in the pink frames are inhibited by SIRT5. PKM2 can be activated and suppressed by SIRT5

Identifying the activity of SIRT5 and its function in disease is a prerequisite for the development of effective inhibitors and agonists. PTMs affect the activity and function of proteins by inducing covalent attachment to new functional groups. PTMs, including acetylation, succinylation, malonylation and glutarylation, are increasingly associated with the regulation of innate immunity, inflammation and metabolism [66]. Studies have shown that SIRT5 regulates the activities of enzymes through PTMs, thus playing an important role in regulating intracellular energy metabolism, signaling pathway transmission and apoptosis [7, 67] (Fig. 4). The research on the regulation of specific substrates by different enzymatic activities of SIRT5 in related diseases are summarized in detail as follows, including the dual role of SIRT5 in tumor.

### Biological functions of SIRT5 deacetylation

Histone acetyltransferase (HAT) mainly regulates specific lysine residues at the amino acid termini of histones, and co-regulates with HDAC to keep gene transcription in a dynamic balance [68]. A total of 277 lysine acetylation sites were identified in 133 mitochondrial proteins, and the functions of the corresponding substrates were mainly focused on the regulation of substances and cancer metabolism [69]. A summary of the SIRT5 deacetylase activity and corresponding substrates in related studies is shown in Table 2.

**Table 2:** Summary of SIRT5 deacetylase activity and corresponding substrates in related studies

Substrate	Site	Activity	Mechanism	Function and disease	References
CPS1	K55, K119, K287	Up	Urea cycle, glycolysis	Hyperammonemia, oocyte quality and fertility	[66]
UOX	–	Up	Urea cycle	Hyperuricemia	[69, 73]
PDX1	–	Down	Inhibit gene transcription	Diabetes	[74]
FOXO3	K271 K290	Up	Apoptosis	Chronic obstructive pulmonary disease	[77]
PML	K487	Up	Redox	Cervical cancer	[79]
Vimentin	K120	Down	Inhibit cell metastasis	Liver cancer	[80]
Cytc	–	Down	Apoptosis	Liver cancer, periodontitis	[81, 86]
GOT1	K369	Down	Redox	Pancreatic ductal adenocarcinoma	[82]
LDHB	K329	Up	Glucose metabolism, apoptosis	Colorectal cancer	[83]
ACAT1	–	Up	Promote cell proliferation	Prostate cancer	[84]
Nrf2/HO-1	–	Up	Redox	Ovarian cancer	[85]
TRIM21	K351	Down	Inhibit gene transcription	Colitis	[87]

### Regulation of metabolism by SIRT5 deacetylation

SIRT5 mainly regulates metabolism, including ammonia metabolism, urea metabolism, and glycolysis. Carbamoyl phosphate synthase 1 (CPS1) is a key rate-limiting enzyme in the urea cycle, condensing ammonia with bicarbonate to form carbamoyl phosphate, which is finally converted into urea and excreted from the kidneys, promoting ammonia removal. As the first substrate found to be deacetylated by SIRT5 [43], CPS1 has a total of 9 lysine (K) acetylation sites, of which K55, K119, and K287 may be key targets [70]. SIRT5 regulates the clearance of ammonia by CPS1 and is regulated by PGC-1α [71], and activation of SIRT5 may contribute to the treatment of hyperammonemia [72]. Urate oxidase (UOX), located in the mitochondria, is a key rate-limiting enzyme in purine catabolism, converting uric acid to allantoin, which is excreted through the kidneys. UOX has at least 10 potential acetylation sites at lysine residues [69] and is upregulated by SIRT5 activity during deacetylation, thereby regulating purine catabolism, with potential for the treatment of hyperuricemia in tumor lysis syndrome [73]. SIRT5 expression is upregulated in pancreatic β-cell lines in patients with type 2 diabetes (T2D), and its expression level is positively correlated with age and blood glucose levels, and negatively correlated with pancreatic and duodenal homeobox 1 (PDX1) expression. SIRT5 deacetylation inhibits PDX1 transcription and insulin secretion, and is a new biomarker for the diagnosis of T2D [74]. However, SIRT5 preserves glucose-stimulated insulin secretion in β-cell lines under glucolipotoxicity [75], which is a good research direction that needs to be further explored in the future.

#### The role of SIRT5 deacetylation in tumor

It has been reported that SIRT5 deacetylation has both positive and negative effects on cancer regulation. Forkhead box O3 (FOXO3) protects cells from oxidative stress by upregulating antioxidants in epithelial cells [76]. SIRT5 deacetylates K271 and K290 of FOXO3, promotes its expression and reduces cigarette smoke extract-induced apoptosis of lung epithelial cells [77]. Unexpectedly, SIRT5 inhibitor suramin and agonist resveratrol exerted the same effect, both promoting the expression of P53 and Bim to induce apoptosis and inhibit the development of cancer [78]. This unique phenomenon may be related to the fact that suramin and resveratrol have multiple targets as natural products and play roles in numerous signaling pathways. However, the molecular mechanism of how suramin and resveratrol synergically regulate SIRT5 and, thus, play a joint role in cancer has not been clarified in detail, and this maybe a special case without generalization. The expression of promyelocytic leukemia protein (PML) is reduced in various cancers, which reduces the sensitivity to hydrogen peroxide and leads to cell death. PML is the first non-mitochondrial protein substrate deacetylated by SIRT5, and its activity is upregulated after being deacetylated at K487, thereby inhibiting hydrogen peroxide-induced proliferation and growth of cervical cancer cells [79]. Vimentin is the main component of the cytoskeleton in eukaryotic cells and associating with cell invasion and poor prognosis. SIRT5 deacetylates K120 of Vimentin, downregulates its activity and inhibits liver cancer cell migration [80]. Cytochrome C (Cytc) is an important electron transporter in the respiratory chain and plays a key role in oxidative metabolism and apoptosis [4]. SIRT5 can deacetylate Cytc, increase the distribution of Cytc in mitochondria to inhibit mitochondrial apoptosis, and is also a potential marker and drug target for the diagnosis and treatment of liver cancer [81]. Aspartate aminotransferase (GOT1) contributes to the maintenance of human pancreatic ductal adenocarcinoma cell (PDAC) by reducing nicotinamide adenine dinucleotide phosphate (NADPH) production. The deacetylation of K369 on GOT1 by SIRT5 downregulates its activity, inhibits glutamine and glutathione metabolism, and exhibits anti-tumor effects on PDAC cells [82].

Although SIRT5 deacetylase activity has been proven to suppress tumor, it is not universally applicable. Lactate dehydrogenase B (LDHB) is a glycolytic enzyme that promotes lysosomal acidification and autophagy in cancer cells. SIRT5 deacetylates K329 of LDHB, upregulates its activity, and promotes autophagy to accelerate colorectal carcinogenesis [83]. This conclusion is consistent with the inhibition of colorectal cancer by SIRT5 modulator mentioned earlier [78]. In addition, SIRT5 enhances the MAPK signaling pathway through acetyl-CoA acetyltransferase 1 (ACAT1), increasing the ability of prostate cancer cells to proliferate, migrate, and invade. This view is also supported by the study that downregulation of SIRT5 leads to significant reductions in cyclin D1 (cell cycle-related) and MMP9 (associated with migration and invasion) [84]. When SIRT5 is overexpressed, the levels of active and functional downstream proteins of MAPK signaling pathway increase, promoting prostate cancer growth. Furthermore, SIRT5 may positively regulate the Nrf2/HO-1 pathway through deacetylation and eliminate cisplatin-induced reactive oxygen species (ROS), thereby inhibiting DNA damage and promoting cell proliferation and resistance in cisplatin ovarian cancer cells [85].

#### SIRT5 deacetylation inhibits inflammation

Inflammation is also affected by SIRT5 deacetylation. SIRT5 inhibits periodontitis by deacetylating Cytc [86]. Lipopolysaccharide-induced polarization of pro-inflammatory macrophages biases tripartite-motif protein (TRIM21)-SIRT5 interactions toward activation of TRIM21 and degradation of SIRT5, leading to increased interleukin-1beta (IL-1β) production in vitro and in vivo, thereby aggravating colitis. SIRT5 deacetylates Lys351 of TRIM21, inhibiting the production of IL-1β and preventing colitis [87]. However, the role of SIRT5 deacetylation in inflammation is still in its infancy and more research is needed.

Although several studies on SIRT5 deacetylation have been reported, some questions remain unanswered. For example, a previous study reported that SIRT5 was involved in regulating the acetylation levels of mouse lens proteins, but its function remained unclear [88]. In addition, HDACs play an important regulatory role in asthma by regulating airway inflammatory infiltration, airway hyper-responsiveness, airway remodeling and maintaining glucocorticoid sensitivity [89]. However, as one of the important members of HDACs, the role of SIRT5 in asthma has not been reported and is worth further study.

### Biological functions of SIRT5 desuccinylation

Protein succinylation refers to the process by which succinyl group donors covalently bind succinyl groups to lysine residues of substrate proteins under enzymatic or non-enzymatic action [90]. After succinylation, lysine carries two negative charges, a conservative modification type that can be specifically recognized by SIRT5 and remove the bound succinyl group to change the properties of the protein [91]. SIRT5 is a global regulator of lysine succinylation in mitochondria, with 2565 succinylation sites identified from 779 proteins in mammals [92]. SIRT5 desuccinylase activity is the most well-studied, focusing on energy metabolism, cancer, cardiac, and neurodegenerative diseases. A summary of SIRT5 desuccinylase activity and corresponding substrates in related studies is shown in Table 3.

**Table 3:** Summary of SIRT5 desuccinylase activity and the role of corresponding substrates in related studies

Substrate	Site	Activity	Mechanism	Disease	References
ACOX1	–	Down	Redox	Liver cancer	[93]
ECHA	K351	Up	Redox	Heart disease	[45]
VLCAD	K482, K492, K507	Up	Metabolism	Fatty acid oxidation	[94]
GLS	K320, K245	Up	Metabolism	Ammonia detoxification	[95]
	K164	Up	Metabolism	Cancer	[98, 99]
HGMCS2	K83, K310	Up	Metabolism	Ketone body production	[96]
PDHA1	K351	Up	Metabolism	Cancer	[44]
SDH	–	Up	Metabolism	Lung cancer, kidney cancer	[92]
IMPDH1	–	Up	Cell proliferation	Cancer	[100]
CS	K393, K395	Up	Cell proliferation	Colon cancer	[101]
SDHA	K547	Down	Metabolism	ccRCC	[102]
IDH2	K413	Up	Redox	ccRCC, heart	[47, 103]
SUN2	–	Down	Cell proliferation	Lung cancer	[6]
p53	K120	Down	Cell repair	Cancer	[104]
LDHA	K118	Down	Cell migration	Prostate cancer	[105]
SHMT2	K280	Up	Redox	Cancer	[106]
OGDH	–	Down	Redox	Stomach cancer	[107]
PKM2	K498	Down	Redox	Colorectal cancer	[110]
	K311E	Up	Anti-inflammation	Colitis	[111]
SOD1	K123	Up	Redox	Lung cancer	[112]
SOD2	–	Up	Redox	Alzheimer’s disease	[113]
ALDH2	K385	Up	Redox	AILI	[114]
PRDX	K84	Down	Redox	Liver ischemia/reperfusion	[116]
OPTN	K108	Up	Autophagy	Diabetic retinopathy	[119]
MAVS	K7	Down	Anti-inflammation	Bacterial infections	[125]
ACOX1	–	Down	Redox	Liver cancer	[126]

#### SIRT5 desuccinylation is involved in energy metabolism

The desuccinylase activity of SIRT5 is involved in regulating key nodes in energy metabolism networks, including fatty acid β-oxidation, branched-chain amino acid catabolism, tricarboxylic acid (TCA) cycle, ketone body synthesis, and glycolysis, and plays a role in disease (Fig. 4). Peroxisomal acyl-CoA oxidase 1 (ACOX1) is the first rate-limiting enzyme in fatty acid β-oxidation, catalyzing the production of hydrogen peroxide (H_2_O_2_), and has been implicated in the development of peroxisomal diseases and liver cancer. SIRT5-mediated desuccinylation inhibits ACOX1 activity and reduces H_2_O_2_ production and oxidative DNA damage [93]. Enoyl-CoA hydratase (ECHA) is also a protein involved in fatty acid oxidation, desuccinylated at K351 and is a main substrate for SIRT5 targeting the heart. When SIRT5 is depleted, ECHA activity is downregulated after excessive succinylation, and cardiac energy metabolism is insufficient, ultimately resulting in cardiomyopathy [45]. Another key enzyme that regulates fatty acid oxidation is very long-chain acyl-CoA dehydrogenase (VLCAD), which can be desuccinylated by SIRT5 at sites K482, K492, and K507, thereby upregulating its activity and promoting fatty acid oxidation [94].

The metabolism of ammonia is closely related to the breakdown of glutamine, and the first critical rate-limiting enzyme for its breakdown is mitochondrial glutaminase (GLS). SIRT5 promotes glutamine metabolism, reduces ammonia accumulation and ammonia-induced mitophagy by desuccinylating Lys 320 and Lys 245 of GLS, upregulating its activity [95]. The rate-limiting ketogenic enzyme 3-hydroxy-3-methylglutaryl-CoA synthase 2 (HMGCS2) desuccinylates K83 and K310, upregulates enzymatic activity, positively regulates ketosis, and reduces acylcarnitine accumulation [96].

The pyruvate dehydrogenase complex (PDC) consists of pyruvate dehydrogenase (E1), dihydrolipoamide transacetylase (E2), and dihydrolipoamide dehydrogenase (E3), the activity of which depends on pyruvate dehydrogenase 1 (PDHA1). SIRT5 desuccinylates at K351 of PDHA1 and increases PDC activity, thereby altering metabolic crosstalk with the TCA cycle, inhibiting the Warburg effect and suppressing tumor progression [44]. Besides, PDC catalyzes the oxidation of pyruvate to acetyl-CoA. Succinate dehydrogenase (SDH) catalyzes the oxidation of succinate to fumaric acid, and at the same time converts ubiquinone to ubiquinol, participating in the TCA cycle. SIRT5 desuccinylation targets the biochemical activity of these two complexes, thereby inhibiting the mitochondrial respiration they regulate [92]. In addition, the *N*-terminal amphipathic helix of SIRT5 has affinity for cardiolipin, and its desuccinylation activity targets protein complexes on the inner mitochondrial membrane, especially complex I, which can also facilitate respiratory chain function [97].

#### The role of SIRT5 desuccinylation in tumor

The role of SIRT5 in diseases, especially cancer, is related to various mechanisms such as metabolism, apoptosis, and redox, and has become an important target for clinical treatment of cancer research. At present, many studies have proved that SIRT5 desuccinylation can promote tumor growth. For example, SIRT5 promotes glutamine metabolism, proliferation, and tumorigenesis by desuccinylation at K164 of the GLS [98, 99]. SIRT5 catalyzes the desuccinylation of inosine-5’-monophosphate dehydrogenase 1 (IMPDH1), which increases its enzymatic activity, thereby enhancing purine biosynthesis and promoting cell proliferation [100]. Citrate synthase (CS), the first rate-limiting enzyme of the TCA cycle, upregulates its activity after desuccinylation by SIRT5 at K393 and K395, promoting colon cancer cell proliferation and migration [101]. SIRT5 desuccinylates the K547 site of succinate dehydrogenase complex flavoprotein subunit A (SDHA), downregulates its activity and inhibits the binding of SDH5, promoting the development of clear cell renal cell carcinoma (ccRCC) occurrence and progress [102]. Another study also demonstrates that desuccinylation increases ccRCC activity. SIRT5 desuccinylates the K413 of isocitrate dehydrogenase 2 (IDH2) and upregulates its activity, leading to increased ccRCC cell viability, increased ROS production and dysfunction of mitochondrial, attenuating the anticancer efficacy of sunitinib [103].

SAD1/UNC84 domain protein-2 (SUN2) is a member of the nuclear membrane LINC (nuclear skeleton-cytoskeletal junction) complex, which inhibits the proliferation and migration of cancer cells and increases the sensitivity of lung cancer cells to cisplatin. However, SIRT5 was found to downregulate the expression of SUN2 in lung cancer [6]. Similarly, as a classic tumor suppressor, p53 repairs damage by inducing cell arrest or eliminates damaged cells through apoptosis. Nonetheless, SIRT5 desuccinylates p53 at K120 and inhibits its activation [104].

Although many articles have proved that SIRT5 desuccinylation can promote tumor growth, there are studies indicating that it can inhibit tumor growth. For example, lactate dehydrogenase A (LDHA) is desuccinylated at K118 by SIRT5, downregulates its activity and inhibits migration and invasion of prostate cancer (PCa) cells [105]. The mitochondrial serine hydroxymethyltransferase 2 (SHMT2) is a key rate-limiting enzyme in serine catabolism and participates in the proliferation of cancer cells. SIRT5 desuccinylates K280 of SHMT2, upregulates its activity, prevents tumor growth, and is a novel mechanism to control cancer cell proliferation [106]. Similarly, desuccinylation inhibits 2-oxoglutarate dehydrogenase (OGDH) activity, decreases mitochondrial membrane potential, reduces ATP production, and increases ROS levels and NADP/NADPH ratio in gastric cancer (GC) cells, thereby inhibiting the growth and migration of GC cells [107, 108]. In addition, SIRT5 desuccinylation inhibits hepatocellular carcinoma (HCC). SIRT5 deficiency results in high degree of succinylation, which increases the production of bile acid, promotes M2-like macrophage polarization, and creates an immunosuppressive tumor microenvironment favorable to tumor-initiating cells [109]. Desuccinylation coordinates bile acid metabolism and prevents tumor immunity from evading, thereby inhibiting HCC.

The desuccinylase activity of SIRT5 is the most complex and active activity. Although much has been studied in tumor, its complex role is still not fully understood, and further research is needed in the future.

#### Antioxidant effect by SIRT5 desuccinylation

The tumor-suppressive mechanism of SIRT5 is also related to antioxidant response. Pyruvate kinase isoform M2 (PKM2) is a key rate-limiting enzyme in the process of glycolysis, which plays a critical role in maintenance redox homeostasis and tumorigenesis. SIRT5 desuccinylates K498 of PKM2, downregulates enzymatic activity, and inhibits cell proliferation and tumor growth stimulated by oxidative stress [110]. Besides, SIRT5 desuccinylates K311E of PKM2, promotes its nuclear entry and binds to the promoter of the IL-1β gene to form a PKM2-HIF1α complex, reduces IL-1β production, and inhibits the pro-inflammatory response of macrophages in colitis [111].

Mitochondrial ROS is the main cause of oxidative stress in cells, while NADPH is the main intracellular reducing agent and plays a key role in the maintenance of reduced glutathione (GSH), which scavenges ROS and thus protects cells from oxidative damage. SIRT5 desuccinylates IDH2 at the K413 site to have antioxidant effects. It upregulates activity and promotes the production of NADPH, thereby increasing the reduced form of GSH and promoting the clearance of ROS [47]. Besides, Cu/Zn SOD1 is a key antioxidant enzyme activated after desuccinylation at K123, which scavenges ROS and inhibits lung tumor cell growth [112]. Similarly, SIRT5 upregulates the activity of the mitochondria-specific antioxidant enzyme manganese SOD2 in MPTP-induced nigrostriatal dopamine antioxidant protection during denaturation [113]. Meanwhile, ROS is a key damage factor in liver diseases. In acetaminophen-induced liver injury (AILI), SIRT5 acts as an antioxidant through the desuccinylation of ALDH2 at the K385 residue, thereby alleviating AILI [114]. Furthermore, it has shown that 2,3,5,4’-tetrahydroxy-stilbene-2-*O*-β-d-glucoside (TSG) inhibits the progression of nonalcoholic fatty liver disease (NAFLD) by increasing the expression of SIRT5, thereby preventing oxidative injury and ameliorating mitochondrial dysfunction, attenuating hepatic steatosis [115]. Unfortunately, the specific mechanism of SIRT5 remains unclear.

Notably, the desuccinylation of SIRT5 seems to play an important role in ischemic injury. Mitochondrial dysfunction is the primary mechanism of liver ischemia/reperfusion (I/R) injury. During I/R, succinylation in liver mitochondria is strongly enriched while the expression of SIRT5 is reduced. Promoting mitochondrial desuccinylation can significantly reduce liver I/R damage. SIRT5 is a key mediator of liver I/R, regulating mitochondrial oxidative stress through desuccinylation of peroxiredoxin-3 (PRDX3) at the K84 site, significantly reducing I/R-induced oxidative damage, apoptosis, and inflammation [116]. Similarly, in subarachnoid hemorrhage (SAH), ischemic exhibits low expression of SIRT5, decreased ATP, and increased production of ROS, leading to neuronal cell death and neurological dysfunction [117]. Therefore, activation of SIRT5 to disrupt lysine succinylation may be a promising therapeutic strategy for SAH.

#### Other effects of SIRT5 desuccinylation

Along with regulating metabolic responses, tumor growth, and redox reactions described above, SIRT5 also has many other functions. Defect in SIRT5 appears to be responsible for ocular disease. Diabetic retinopathy is a common microvascular complication of diabetes mellitus [118]. SIRT5-mediated desuccinylation of optineurin (OPTN) at K108 reverses high glucose-induced dysfunction of autophagic flux, reduces the loss of retinal ganglion cells, and protects their function [119]. Meanwhile, SIRT1, 3, 5, and 6 are considered to be regulators of diabetic retinopathy since they control the sensitivity to insulin, as well as the initiation of glycolysis, gluconeogenesis, and inflammatory processes [120].

SIRT5 exhibits the maintenance of cardiac function in response to pressure overload. *Sirt5* KO mice are more susceptible to injury and dysfunction after cardiac stress, but the mechanism cannot be determined and it can only speculate that SIRT5 overexpression may prevent hypertrophy and heart failure by regulating levels of succinic acid and other metabolites [121]. Fortunately, we may be able to start with quercetin, an agonist of SIRT5. Studies have shown that quercetin can promote the desuccinylation of IDH2 through SIRT5, maintain mitochondrial homeostasis, protect mouse cardiomyocytes under inflammatory conditions, and improve myocardial fibrosis [122, 123], to reduce the incidence of heart failure. Besides, quercetin can also inhibit non-small cell lung cancer via SIRT5 [124]. They found that quercetin can activate the SIRT5, inhibit the phosphorylation of p-PI3K and p-AKT which reduce DNA damage repair (DDR) to raise DNA damage and apoptosis in NSCLC.

Mitochondrial dysfunction plays an important role in the development of intervertebral disc degeneration (IDD). Excessive mechanical load increases the level of succinylation of AIFM1 in nucleus pulposus cells by reducing the expression of SIRT5, thereby eliminating the interaction between AIFM1 and CHCHD4, leading to mitochondrial dysfunction and ultimately developing into IDD [125]. Unfortunately, current treatments for IDD aim to relieve pain and other symptoms, preventing IDD remains a key challenge.

The mitochondrial antiviral signaling protein (MAVS) is a key factor in RLR signaling in the innate antiviral immune response. Desuccinylation by SIRT5 at the K7 site reduces the formation of MAVS aggregates after viral infection, leading to inhibition of MAVS activation and downregulation of the body’s antiviral capacity [126]. Therefore, we can preliminarily conclude that SIRT5 promotes viral replication. However, whether this conclusion is correct or not remains to be further confirmed. An article reports that SARS-CoV-2 infection interacts with SIRT5 through a viral non-structural protein (NSP14), thus promoting succinylation of several key enzymes in TCA, inhibiting cellular metabolic pathways and promoting viral replication [127]. Conversely, another study states that SIRT5 is a proviral factor necessary for viral replication [128].

Desuccinylation is ubiquitous in biology and is widely involved in various biological processes such as metabolic regulation, epigenetic regulation, and signal transduction. As an important desuccinylase, SIRT5 not only promotes tumor proliferation and metastasis, but also inhibits the growth and apoptosis of cancer cells. Therefore, it is necessary to develop selective modulators of SIRT5 to provide new avenues for the treatment of diseases, especially cancer.

### Biological functions of SIRT5 demalonylation

SIRT5 has a role in removing malonyl groups from proteins. Recently, 1,137 sites in 430 proteins were found to combine with malonyl groups. Only 44% of the sites bind to malonyl groups, while the remaining 56% can also bind to succinyl groups, which are mainly enriched in the glycolytic pathway [46]. A summary of SIRT5 demalonylation activities and corresponding substrates in related studies is shown in Table 4.

**Table 4:** Summary of SIRT5 demalonylase activity and the role of corresponding substrates in related studies

Substrate	Site	Activity	Mechanism	Function and disease	References
GAPDH	K184	Up	Metabolism	Glycolysis	[46]
GSTP1	K121	Down	Antioxidant	Diabetic cardiomyopathy	[130]
SDHA	K179	Down	Metabolism	Colorectal cancer	[132]
TKT	—	Up	Metabolism	Colorectal cancer	[133]
DDX3	K66, K130, K162	Down	Gene transcription	Viral infection	[134]

The demalonylation activity of SIRT5 mainly focuses on the glycolysis/gluconeogenesis pathway. Malonyl-CoA is elevated in skeletal muscle and liver from obese rats and in muscle biopsies from obese and T2D patients, and liver overexpression of SIRT5 enhances glycolysis in hepatocytes and inhibits gluconeogenesis, reducing hepatic triglycerides content of hepatic steatosis [129]. In addition, in diabetic cardiomyopathy mice, SIRT5 was found to demalonylate the K121 site of glutathione S-transferase P 1 (GSTP1), thereby exerting antioxidant protection [130]. SIRT5 demalonylates the K184 site of the key glycolysis enzyme GAPDH, upregulating its activity and reducing the shift of glucose from oxidation in glycolysis to glycogen synthesis or the pentose phosphate pathway [46]. Methylmalonic acidemia (MMA) is usually caused by defects in amino acid and fatty acid catabolism. Methylmalonylation inhibits enzymes in the urea cycle and glycine cleavage pathway in MMA, while SIRT5 demalonylase activity effectively ameliorates hyperammonemia in MMA mice [131].

SIRT5 is not only involved in physiological metabolism, but also in tumor regulation and immunomodulation. SIRT5 demalonylates K179 of SDHA, downregulating its activity, leading to succinate accumulation and activation of the ROS scavenging enzyme thioredoxin reductase 2 (TrxR2), causing colon cancer cell resistance to rituximab [132]. Another study finds that SIRT5 activates the key enzyme transketolase (TKT) in the non-oxidized pentose phosphate pathway in a demalonylation-dependent manner, maintaining sufficient nucleotide levels for DNA synthesis and promoting colorectal cancer growth. Consequently, inhibition of demalonylase activity of SIRT5 is a potential therapeutic pathway for colorectal cancer [133]. DDX3 is a member of the DEAD (Asp-Glu-Ala-Asp) box containing the ATPase-dependent RNA helicase family. Malonylation at K66, K130, and K162 is inhibited by SIRT5, which increases TBK1 signaling and enhances the body’s antiviral innate immune response [134].

The research of SIRT5 demalonylase activity is still in its infancy and has only been clarified in a few diseases. One of the reasons for its unclear function in vivo may be the lack of insight into protein and lysine residue modifications. Malonyl-CoA is the cornerstone of nascent fatty acid synthesis and a critical pathway for regulating cell function and survival [135]. Hyperglycemia causes excessive malonylation by elevating malonyl-CoA levels and accumulating in non-lipid tissues such as heart and skeletal muscle [136]. The prevalence and potential pathophysiological outcomes of chronic hypermalonylation modifications in these tissues are unclear. It is necessary to explore whether the demalonylase activity of SIRT5 ameliorates adverse outcomes. Besides, the metabolism of glucose and fatty acids in liver tissues is regulated by lysine demalonylation, suggesting that the occurrence of metabolic diseases such as obesity may be associated with demalonylation [137]. Therefore, demalonylation of SIRT5 may provide new insights into cellular energy regulation in vivo and further elucidates the role of SIRT5 in systemic metabolic diseases. Interestingly, the demalonylation sites of SIRT5-targeted substrates overlap with deacetylation and desuccinylation sites, especially key enzymes involved in glycolysis and urea cycling. It would be worthwhile to further investigate how SIRT5 coordinates these different biological functional pathways.

### Biological functions of SIRT5 deglutarylation

Glutarylation is a widely conserved PTMs in eukaryotic and prokaryotic cells, as well as the process of covalently binding glutaryl groups to a lysine residue of substrate proteins catalyzed by enzymes [138]. SIRT5 is the only HDAC capable of acting as lysine deglutarylase in vitro and in vivo, with 683 glutarylated lysine sites identified in 191 proteins [59]. A summary of SIRT5 deglutarylation activities and corresponding substrates in related studies is shown in Table 5.

**Table 5:** Summary of SIRT5 deglutarylation activity and corresponding substrates in related research

Substrate	Site	Activity	Mechanism	Function and disease	References
CPS1	K55, K219, K412, K889, K892, K915, K1360, K1486	Up	Ammonia metabolism	Glutaric acidemia type I	[59]
G6PD	—	Up	Redox	Clear ROS	[47]
GLUD l	K54	Up	Metabolism	Colorectal cancer	[139]

The key rate-limiting enzyme in the urea cycle, CPS1, is essential for ammonia detoxification. Eight SIRT5 deglutarylation sites were identified in CPS1, including K55, K219, K412, K889, K892, K915, K1360, K1486. After deglutarylation of CPS1, its activity is upregulated and may reduce ammonia levels in type I glutaric acidemia [59]. SIRT5 in mitochondria can promote the deglutarylation of glucose-6-phosphate dehydrogenase (G6PD), promote the production of NADPH and GSH, and scavenge ROS to prevent oxidative stress damage [105]. SIRT5 deglutarylates glutamate dehydrogenase 1 (GLUD l) at K545 site and activates its activity, promotes glutamine synthesis and TCA cycling, and promotes colorectal cancer cell growth and proliferation [139]. SIRT5 may regulate the level of intracellular glutarylation to control the differentiation of brown adipocyte, and indirectly affect the activation of brown fat genes, providing a new direction for the treatment of obesity [140].

Deglutarylation is a NAD^+^-mediated PTM of proteins regulated by SIRT5, the discovery of which further expands the functionality of SIRT5. The substrate enrichment results of SIRT5 show that it is primarily localized in mitochondria and involved in the oxidative metabolic pathway. This indicates that the deglutarylation modification of SIRT5 may be involved in the regulation of various cellular enzymatic processes and is closely related to mitochondrial metabolism. Combined with the fundamental role of mitochondria in providing energy and regulating cellular metabolic homeostasis, whether the deglutarylation of SIRT5 is involved in the regulation of diseases associated with mitochondrial dysfunction remains to be investigated. In conclusion, the effect of SIRT5 regulation of deglutarylation on disease remains a mystery. Future studies are required to investigate other substrates recognized by SIRT5 deglutarylation and reveal the role of these substrates in disease.

## Natural agonists and inhibitors of SIRT5

As a potential therapeutic target for a variety of diseases, many studies have been conducted on the alteration of SIRT5 activity over the years. More synthetic compounds that regulate SIRT5 can be found in previously published paper [141], while natural compounds are ignored. Here we list some potential natural compounds to draw attention to natural compounds as regulators of SIRT5. We also discussed some techniques and methods regarding the mining of SIRT5 modulators. We hope that in the future, some researchers will be able to develop more potent and selective modulators based on the structures of these natural modulators.

Resveratrol is one of the most classic agonists of the sirtuin family, which can delay aging and reduce inflammation and apoptosis of the vascular endothelium [142]. Resveratrol can also stimulate the expression of SIRT5 in SAH, thereby inducing desuccinylation of citrate synthase (CS) and ATP synthase, which can effectively improve mitochondrial metabolic function and reduce brain damage [117]. As previously reported, quercetin stimulated SIRT5 expression, inhibited oxidative stress damage and inflammation, maintained cardiomyocyte activity, and reduced myocardial fibrosis damage in mice with heart failure [122]. Since sirtuins are a family of NAD^+^-dependent enzymes, sirtuins transfers acyl groups from the lysine side chain to ADP ribose, which is formed by the co-substrate NAD through the release of nicotinamide [17] (Fig. 3). Therefore, nicotinamide is considered to be a general sirtuins inhibitor, which can inhibit the expression of SIRT5 and weaken its activity (Table 6). Recent studies have confirmed that nicotinamide riboside (NR), as a precursor to NAD^+^, is able to selectively activate the deacetylase activity of SIRT5 instead of the other enzyme activities [143].

**Table 6:**
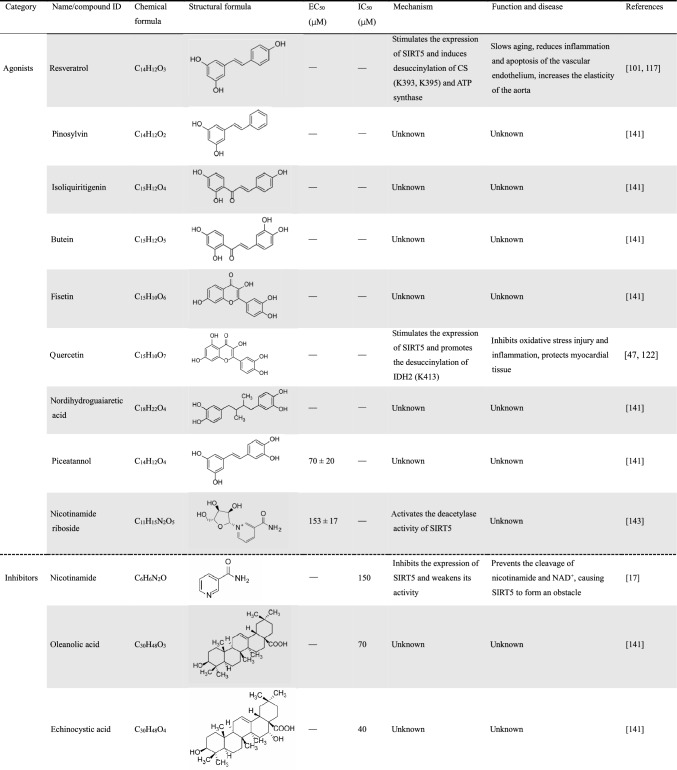

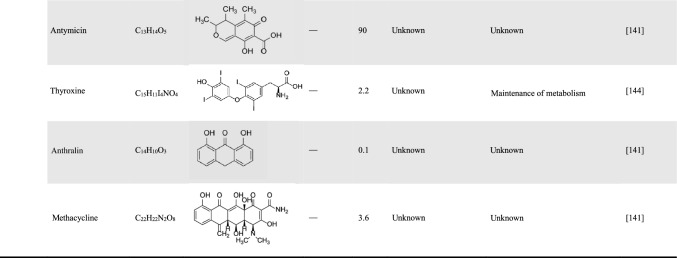
Natural agonists and inhibitors of SIRT5 and their roles in related studies

The crystal structure formed by the interaction of SIRT5 with its substrate and NAD^+^ is a breakthrough point for the development and design of new inhibitors and agonists of SIRT5 [145]. Targeting substrate-binding sites is a more efficient strategy than targeting NAD^+^, a common substrate for most human enzymes, which has limited selectivity. Indeed, a great deal of work has been carried out on inhibitor design approaches based on the SIRT5 structure and promising peptide inhibitors have been developed, providing vital information for further drug mining [146]. Six potent peptidyl inhibitors that interact with the NAD^+^ binding pocket were discovered by modifying the lysine side chain based on a CPS1-derived peptide substrate [147]. These X-ray crystal structures reveal SIRT5 acyl selectivity and its molecular basis, which enable the design of SIRT5 inhibitors. Specifically, rapid overlay of chemical structures (ROCS), ShaEP, and Phase Shape can be used to identify small-molecule compounds with similar shapes to SIRT5 substrates based on the principle of three-dimensional (3D) structure [148, 149]. In addition, the pharmacophore method can be used to generate rational pharmacophore models based on the structure of substrate-SIRT5 to find potential inhibitors [150]. At the same time, molecular docking techniques and molecular dynamics methods are also very useful [151].

Although the structure of SIRT5 has commonalities with other sirtuins, the low specificity of current SIRT5 regulators means that they can act on other sirtuins as well. Actually, SIRT5 has its own unique structural features. Ala86 is unique to SIRT5, making the substrate-binding pocket of SIRT5 much larger than other sirtuins, thus allowing interaction with bulkier acylated lysine substrates. Besides, SIRT5 has two specific non-hydrophobic amino acids, Tyr102 and Arg105, that define its substrate selectivity and enzymatic activity. Mechanistically, these two amino acids locate deep within the substrate-binding site and interact with the negatively charged acyl-lysine substrate through hydrogen bonding and electrostatic interactions [7]. Even so, SIRT5 has multiple roles in disease development, making it difficult for SIRT5 regulators to work precisely. Therefore, the role of SIRT5 in disease should also be considered when exploring SIRT5-specific regulators. Perhaps in the future, we can target specific SIRT5 isoform, such as SIRT5^iso2^, which is almost exclusively expressed and may play an important role in the brain [9]. In conclusion, searching effective inhibitors or agonists based on the specific structure of SIRT5 and its role in disease may be the focus of future research.

## Potential chemicals acting on SIRT5 based on virtual docking

**Fig. 5:**
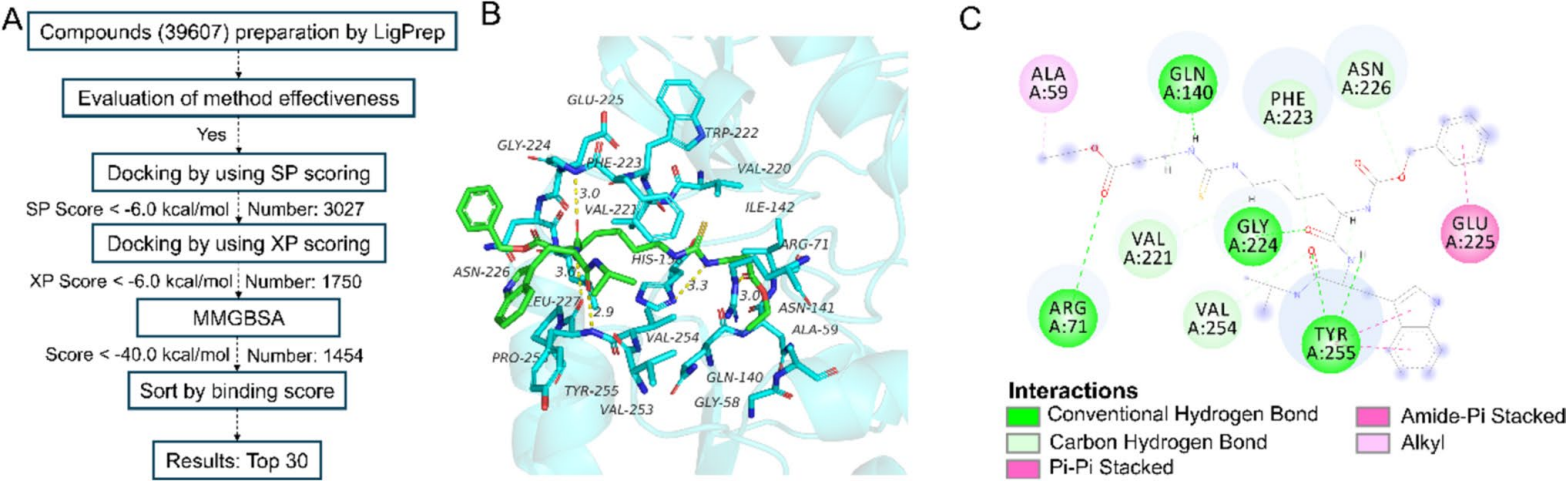

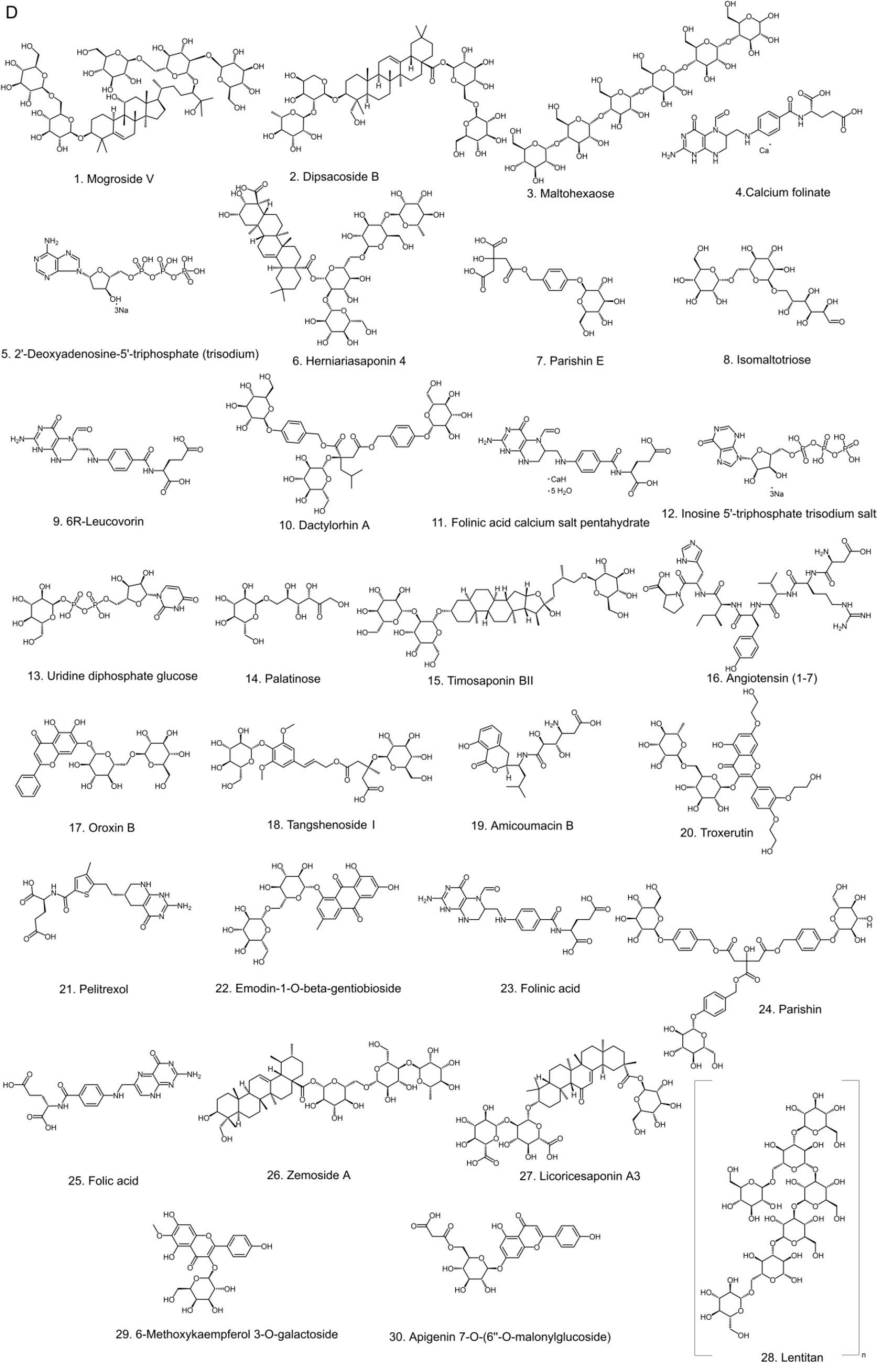
Potential chemicals acting on SIRT5 based on virtual docking. **A** A workflow of virtual screening. **B** A close view of the active site binding with ligand. Key residues interacted with ligand were rendered in stick and colored by cyan. **C** The 2D protein–ligand interaction diagram of ligand-Sirt5 complex. **D** Chemical structure information of the top 30 compounds obtained based on docking research

We conducted docking studies on the SIRT5 target with 39,607 compounds sourced primarily from FDA-approved and Pharmacopoeia Drug Libraries (TargetMol, L1010), Natural Product Library (Topscience, L6020), and Bioactive Compound Library (MedChemExpress, HY-L001). We selected the crystal structure of the SIRT5 protein (PDB number: 3RIY), which was pre-processed and then subjected to docking calculations using the method illustrated in the figure (Schrödinger Maestro, Fig. 5A) to screen for potential key active components. We also analyzed the binding modes and the validity of the method, using the SIRT5 positive compound Et-29 as a reference. The results indicated that Et-29 could form strong hydrogen bonds with the key residues Gln-140, Gly-224, Arg-71, and Tyr-255 of Sirt5, while the phenyl ring could engage in pi–pi and pi–amide conjugated interactions with the Tyr-255 and Glu-225 amino acids, respectively (Fig. 5B-C). These interactions play a crucial role in stabilizing the ligand, demonstrating that this screening method is effective and reasonable. Based on the above approach, we docked the 39,607 compounds and identified 1,454 potentially active compounds (SP score < -6.0 kcal/mol, XP score < -6.0 kcal/mol, molecular mechanics generalized born surface area (MM/GBSA) score < -40.0 kcal/mol, Table S1-S3). Further evaluation using MM/GBSA and binding energy scoring methods yielded the top 30 compounds (Fig. 5D, Table 7).

**Table 7:** Top 30 compounds obtained by virtual screening

No	Compound	CAS	Docking score	Log P	Molecular weight
1	Mogroside V	88,901–36-4	-13.45	-4.56	1287.45
2	Dipsacoside B	33,289–85-9	-13.30	-1.39	1075.25
3	Maltohexaose	34,620–77-4	-12.95	-12.52	990.86
4	Calcium folinate	1492–18-8	-12.55	-1.17	473.45
5	2’-Deoxyadenosine-5’-triphosphate (trisodium)	54,680–12-5	-12.29	-3.95	491.18
6	Herniariasaponin 4	177,263–83-1	-12.28	-3.17	1135.26
7	Parishin E	952,068–57-4	-12.28	-2.05	460.39
8	Isomaltotriose	3371–50-4	-12.27	-6.77	504.44
9	6R-Leucovorin	73,951–54-9	-12.14	-1.14	473.45
10	Dactylorhin A	256,459–34-4	-11.98	-2.57	888.87
11	Folinic acid calcium salt pentahydrate	6035–45-6	-11.89	-1.17	473.45
12	Inosine 5’-triphosphate trisodium salt	35,908–31-7	-11.84	-4.67	508.17
13	Uridine diphosphate glucose	133–89-1	-11.83	-6.70	566.30
14	Palatinose	13,718–94-0	-11.83	-5.18	342.30
15	Timosaponin BII	136,656–07-0	-11.75	0.24	921.08
16	Angiotensin (1–7)	51,833–78-4	-11.73	-0.78	899.02
17	Oroxin B	114,482–86-9	-11.71	-1.69	594.52
18	Tangshenoside I	117,278–74-7	-11.62	-3.00	678.64
19	Amicoumacin B	82,768–33-0	-11.55	0.22	424.45
20	Troxerutin	7085–55-4	-11.47	-2.65	742.68
21	Pelitrexol	446,022–33-9	-11.47	0.35	463.51
22	Emodin-1-*O*-β-gentiobioside	849,789–95-3	-11.44	-1.70	594.52
23	Folinic acid	58–05-9	-11.43	-1.14	473.45
24	Parishin	62,499–28-9	-11.35	-3.09	996.92
25	Folic acid	59–30-3	-11.34	-0.30	441.40
26	Zemoside A	593,254–83-2	-11.32	0.16	943.13
27	Licoricesaponin A3	118,325–22-7	-11.30	-0.35	985.08
28	Lentinan	37,339–90-5	-11.28	-14.43	1153.00
29	6-Methoxykaempferol 3-*O*-galactoside	72,945–43-8	-11.24	-0.25	954.80
30	Apigenin 7-*O*-(6’’-*O*-malonylglucoside)	86,546–87-4	-11.22	0.33	518.43

## Conclusion

Mitochondria are the centers of metabolism, controlling all aspects of cellular and organismal physiology, and are essential to maintain cell homeostasis, development, aging, and apoptosis [152]. Therefore, changes in mitochondria lead to alterations in gene expression and protein modification. Targeting metabolic control with various compounds can delay or treat associated diseases. The need for effective modulators of SIRT5 is increasing as more and more studies associate SIRT5 with a range of diseases, particularly cancer. Hence, there is a great potential for treating cancer and other illnesses by elucidating the interrelationship between SIRT5 and metabolism. Research on the function of SIRT5 in metabolic processes is crucial for metabolic regulation and greater comprehension of its role in physiological and pathological settings. SIRT5 has complex functions, and in the context of cancer, it exhibits a contradictory role. On the one hand, it acts as a tumor suppressor to inhibit cancer, and on the other hand, it acts as an oncogene to activate cancer, depending on various factors [7, 153]. Besides, the role of SIRT5 in viral transcription and replication has not been well-studied. Clarifying whether SIRT5 plays a positive or negative role in viral replication and the interaction between SARs and SIRT5 is an important direction for future research. The current findings suggest that SIRT5 may be a potential drug target against COVID-19.

With its diverse range of functions, SIRT5 presents a potential target for developing agonists or inhibitors that can serve as effective therapeutic agents for an array of illnesses, including cancer, cardiovascular disease, and neurodegeneration. Furthermore, researchers may gain a deeper understanding of the physiological and pathological environment by studying the selective regulation of SIRT5. Nevertheless, the research of this modulator is still in its infancy, but the availability of SIRT5 crystal structures suggests that a structure-based approach to drug design is possible. Here, we present the structural features of SIRT5 and provide a detailed summary of the currently recognized substrates responsible for the four enzymatic activities of SIRT5, as well as their impact on illness. In addition, we outline some natural modulators of SIRT5 and review the internship between various SIRT5 enzymatic activities and metabolism. More importantly, we found thousands of potential targets that can be acted upon by SIRT5 through virtual docking technology. Here we list the top 30 compounds with the highest relevance, and we hope that more studies will be based on them in the future. We also hope that this review will deepen the understanding of SIRT5 and provide useful information for the further development of novel SIRT5 regulators for the treatment of related diseases. Although the exact mechanism of SIRT5 enzymatic activity in most diseases remains unknown and studies of SIRT5 demalonylation and deglutarylation enzymatic activity are still in their infancy, we still believe that targeting SIRT5 and/or SIRT5-binding substrates for diseases treatment based on the unique structure of SIRT5 presents a highly promising therapeutic approach.

## Supplementary Information

Below is the link to the electronic supplementary material.

Supplementary file1 (CSV 181 KB)

Supplementary file2 (CSV 1246 KB)

Supplementary file3 (CSV 978 KB)
